# Amyloid precursor protein modulates Nav1.6 sodium channel currents through a Go-coupled JNK pathway

**DOI:** 10.1038/srep39320

**Published:** 2016-12-23

**Authors:** Shao Li, Xi Wang, Quan-Hong Ma, Wu-lin Yang, Xiao-Gang Zhang, Gavin S. Dawe, Zhi-Cheng Xiao

**Affiliations:** 1 Department of Physiology, Dalian Medical University, Dalian 116044, China; 2 Department of Pharmacology, Yong Loo Lin School of Medicine, National University Health System, National University of Singapore, 10 Medical Drive 117597, Singapore; 3 The Key Laboratory of Stem Cell and Regenerative Medicine, Institute of Molecular and Clinical Medicine, Kunming Medical College, Kunming 650031, China; 4 Jiangsu Key Laboratory of Translational Research and Therapy for Neuro-Psycho-Diseases, Institute of Neuroscience, Second Affiliated Hospital, Soochow University, Suzhou, Jiangsu Province 215123, China; 5 Neurobiology and Ageing Programme, Life Sciences Institute, Centre for Life Sciences, National University of Singapore, 28 Medical Drive 117456, Singapore; 6 Singapore Institute for Neurotechnology (SINAPSE), Centre for Life Sciences, National University of Singapore, 28 Medical Drive 117456, Singapore; 7 Department of Anatomy and Developmental Biology, Monash University, Clayton, Melbourne, Victoria 3800, Australia

## Abstract

Amyloid precursor protein (APP), commonly associated with Alzheimer’s disease, also marks axonal degeneration. In the recent studies, we demonstrated that APP aggregated at nodes of Ranvier (NORs) in myelinated central nervous system (CNS) axons and interacted with Nav1.6. However, the physiological function of APP remains unknown. In this study, we described reduced sodium current densities in APP knockout hippocampal neurons. Coexpression of APP or its intracellular domains containing a VTPEER motif with Na_v_1.6 sodium channels in Xenopus oocytes resulted in an increase in peak sodium currents, which was enhanced by constitutively active Go mutant and blocked by a dominant negative mutant. JNK and CDK5 inhibitor attenuated increases in Nav1.6 sodium currents induced by overexpression of APP. Nav1.6 sodium currents were increased by APPT668E (mutant Thr to Glu) and decreased by T668A (mutant Thr to ALa) mutant, respectively. The cell surface expression of Nav1.6 sodium channels in the white matter of spinal cord and the spinal conduction velocity is decreased in APP, p35 and JNK3 knockout mice. Therefore, APP modulates Nav1.6 sodium channels through a Go-coupled JNK pathway, which is dependent on phosphorylation of APP at Thr668.

Amyloid precursor protein (APP) is well known for its function in the pathogenesis of neurodegenerative disorders, such as Alzheimer’s disease (AD). Abnormalities in proteolytic processing of APP lead to generation of amyloidogenic peptides that are involved in the pathological presentation of amyloid plaques, an initiator in AD pathogenesis. APP has long been utilized as a marker for axonal degeneration following neural injury and aggregated at nodes of Ranvier (NORs) in the normal myelinated axons^1^, but its physiological function along myelinated axons has yet to be elucidated. Increasing evidence strongly suggests that an important component of AD is damage to myelin^2^,^3^. AD patients have an increased incidence of epileptic seizures, especially in patients with early-onset APP who overexpress mutant human APP^4^, suggesting that APP may modulate neuronal excitability^5^. Moreover, APP knockout (KO) mice exhibit decreased locomotor activity and forelimb grip strength^6^, implicating abnormalities related to myelination and saltatory conduction along myelinated axons. However, the mechanisms by which APP relates to these phenotypes remain unknown.

Our previous study demonstrates that APP aggregates at NORs in myelinated central nervous system (CNS) axons, but not in the peripheral nervous system (PNS)^1^. The establishment and maintenance of the elaborated architecture of axonal domains and the congregation of specific ion channels and recognition molecules, such as Nav1.6 sodium channels, F3/contactin, tenascin-R, and OMgp, at the NORs are critical to ensure rapid saltatory conduction of action potentials along myelinated fibers^7^. Voltage-dependent sodium channels are pores comprised of one α-subunit and one or more auxiliary β-subunits. An α subunit forms the core of the channel and is functional on its own. When the α subunit protein is expressed by a cell, it is able to form a channel that conduct Na^+^ in a voltage-gated way, even if β subunits or other known modulating proteins are not expressed. Four distinct α-subunits of sodium channels are expressed in mammalian CNS neurons. They are Nav1.1, Nav1.2, Nav1.3, and Nav1.6^8^. Among them, TTX-sensitive Nav1.2 and Nav1.6 are uniformly distributed along unmyelinated axons, but are clustered at the NORs in myelinated axons. During development, Nav1.2 is expressed and clusters at immature NOR, but is replaced by Nav1.6 at mature NOR, which might allow neurons to adapt to high-frequency firing^8^. The fine modulation of sodium channels, responsible for depolarizing and repolarizing events at the NORs, is essential for precise saltatory conduction along myelinated axons.

An understanding of how APP is associated with myelinated axons is important, as myelination is a vital biological process disrupted in a wide variety of congenital and acquired neurological diseases, including AD. Thus, according to the phenotype of APP KO mice, we hypothesize that APP, which takes on a characteristic distribution along axons, will render it a candidate molecule for ion channel modulation. We recently reporte that APP colocalizes and interactes with Nav1.6 in mouse cortical neurons^9^. In the present study, we show that APP positively modulates sodium currents of Nav1.6 in an APP Thr668 phosphorylation-dependent manner through a Go-coupled JNK pathway.

## Results

### Conduction velocities of compound action potentials are reduced in the spinal cords of APP knockout mice

**Figure 1:**
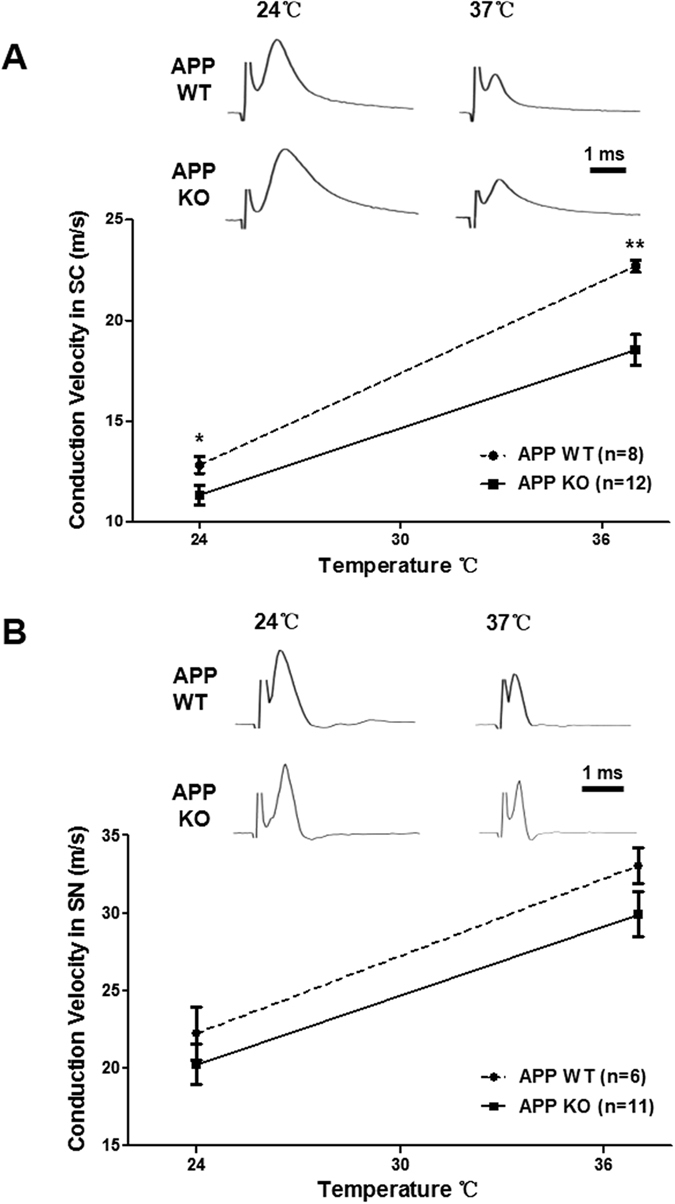
Conduction velocities of compound action potentials (CAPs) are reduced in the spinal cords of APP KO mice. Representative CAPs from the spinal cords (SC, **A**) and the sciatic nerve (SN, **B**) of APP KO and WT mice. Representative CAPs recorded at 24 °C and 37 °C from spinal cords of APP KO and WT mice (upper panel). The conduction velocity measured in WT mouse spinal cord (open bars) was consistently higher than that seen in APP KO mice (closed bars) at both temperatures measured (lower panel). N numbers are indicated inside the bars. Student’s t-test, *p < 0.05; **p < 0.01. Error bars represent mean ± S.E.

APP KO mice exhibit decreased locomotor activity and forelimb grip strength^6^, suggesting functional abnormalities in the myelinated axons.We thus recorded compound action potentials (CAPs) of APP KO and littermate wild-type (WT) mice. We used a double grease-gap chamber (fixed conduction distance) and suction electrode^10^ to measure CAPs from spinal cord and sciatic nerve, respectively. The time-to-peak for each trace was then measured and converted to a conduction velocity. The average conduction velocities in the spinal cords of APP KO mice were lower than those in WT mice at both 24 °C and 37 °C (At 24 °C, WT: 12.83 ± 0.42 ms^−1^, APP KO: 11.34 ± 0.49 ms^−1^, *p* < 0.05; At 37 °C, WT: 22.69 ± 0.30 ms^−1^, APP KO: 18.53 ± 0.77 ms^−1^, *p* < 0.005; Fig. 1A). However, the conduction velocities in the sciatic nerves of APP KO mice showed comparable levels to those in WT mice at both 24 °C and 37 °C (At 24 °C, WT: 22.23 ± 1.71 ms^−1^, APP KO: 20.24 ± 1.31 ms^−1^; At 37 °C, WT: 33.05 ± 2.13 ms^−1^, KO: 29.93 ± 1.48 ms^−1^; Fig. 1B). These results implies that APP plays a role in modulating the conduction of action potentials in CNS, but not in PNS.

### APP enhances sodium current density through modulation of α-subunit of Nav1.6

**Figure 2:**
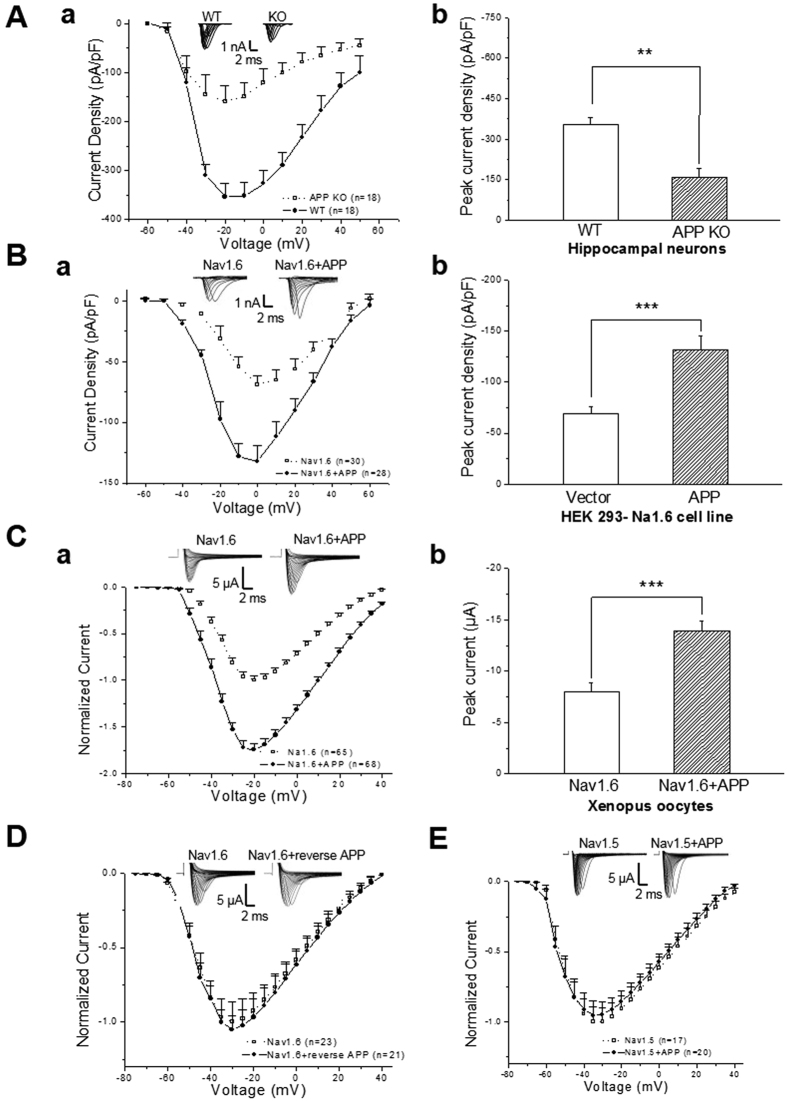
APP enhances of sodium current expression through modulation of α-subunit of Nav1.6. (**A**) Sodium current expression is reduced in hippocampal neurons from APP KO mouse. Whole cell recordings of voltage-gated sodium currents from hippocampal slice of APP KO and WT mice at 7–8 weeks old of age. Sodium current responses elicited by depolarizing voltage steps (from −60 mV to 60 mV, 10 mV increments) in a neuron from WT and APP KO mice. (a), Current-voltage relationships of sodium currents density in WT mice (open squares) and APP KO mice (solid circles). pF, picofarads. (b) Peak currents density in APP KO and their littermate WT mice. (**B**) Overexpression of APP enhances sodium currents in HEK293 cells stably expressing α-subunit of human Nav1.6 (HEK293-Nav1.6 cells). Whole cell recordings of voltage-gated sodium currents in APP- and empty vector (vector)-transfected HEK293-Nav1.6 cells. Sodium current responses elicited by depolarizing voltage steps (from −60 mV to 60 mV, 10 mV increments). (a), Current-voltage relationships of sodium currents density in vector-(open squares) and APP-transfected HEK293-Nav1.6 cells (closed circles). pF, picofarads. (b), Peak currents density in vector- and APP-transfected HEK293-Nav1.6 cells. (**C**) APP enhances sodium currents in Xenopus oocytes. Two-electrode voltage-clamp recordings in oocytes which were injected cRNA for Nav1.6 α subunits and together with APP. The oocytes injected cRNA for Nav1.6 α subunits alone were recorded as the control. Sodium current responses elicited by depolarizing voltage steps (−75 mV to 40 mV, 5 mV increments). (a), Normalized current-voltage relationships of currents in oocytes expressing Nav1.6 α subunits alone (open squares) and together with APP (closed circles). (b), Sodium peak currents in oocytes expressing Nav1.6 α subunits alone and together with APP. (**D**) Normalized current-voltage relationships of sodium currents by two-electrode voltage-clamp recordings in oocytes injected cRNA for Nav1.6 α subunit alone (open squares) and those injected cRNA for Nav1.6 α subunitvand the reversed sequence of APP (closed circles). (**E**) Normalized current-voltage relationships of sodium currents by two-electrode voltage-clamp recordings in oocytes expressing Nav1.5 α subunit alone (open squares) and those expressing Nav1.5 α subunit together with APP (closed circles). Insert: Representative examples of sodium current responses elicited by depolarizing voltage steps. One-way ANOVA, *p < 0.05; **p < 0.01; ***p < 0.001. Error bars represent mean ± S.E.

Given that sodium channels play essential roles in initiation and conduction of action potentials, we hypothesized that APP might modulate the function of sodium channels. To investigate the effects of APP on sodium currents, we performed whole cell recordings from hippocampal neurons of brain slices in APP KO and WT mice. After pharmacological suppression of voltage-dependent calcium and potassium currents with Cd^2+^ and Cs^+^, respectively, fast sodium currents were activated by stepped depolarizations. To determine the current-voltage (I-V) relationship of the sodium current, the peak current amplitude at each voltage step was normalized to the neuron’s capacitance and plotted as a function of the command potential. As capacitance is related to cell size, correction for capacitance in this manner should compensate for changes attributable to difference in cell size. The peak sodium current densities in hippocampal neurons decreased in APP KO compared to those in WT mice as shown by I-V curve of sodium currents (Fig. 2Aa) and the peak currents density (Fig. 2Ab), suggestig that APP enhances sodium current density in mammalian cells.

CNS neurons express Nav1.1, Nav1.2, Nav1.3, and Nav1.6. Among them, Nav1.6 is the predominant α subunit of sodium channel locating at CNS NORs and is required for conduction of action potentials^11^. Considering that APP colocalizes with Nav1.6 at the NORs along adult CNS myelinated axons and in the soma and proximal neuritis of cultured primay cortical neurons^1^,^9^, we were wondering whether APP modulated Nav1.6 currents. We recorded Nav1.6 currents in HEK293 cells stably expressing human Nav1.6 (HEK293-Nav1.6)^12^, which have been transfected with full-length APP. Sodium current densities were significantly increased in APP-transfected HEK293-Nav1.6 cells compared with those in control cells, which were transfected with the empty vector (Fig. 2Ba,b). In contrast, sodium current densities remain unchanged in HEK293-Nav1.2 cells which stably express human Nav1.2, upon transfection of APP (Fig. S1), indicating that APP does not modulate Nav1.2. Therefore, these results indicate that APP increases Nav1.6 sodium current density in mammalian cells.

β1 and β2 subunits of sodium channels are expressed endogenously in both HEK293 cells and cultured hippocampal neurons^13^,^14^,^15^,^16^. To investigate whether APP enhancecs sodium current density through modulation of either α subunit or β1/β2 subunit of Nav1.6, we co-expressed full-length APP with α subunit of Nav1.6 in Xenopus laevis oocytes, where few β1, β2 and α subunits of Nav1.6 are expressed endogenously. Two-electrode voltage-clamp recordings were performed for sodium currents. Greater sodium currents were recorded in the oocytes co-injected with cRNAs forα subunit of Nav1.6 and APP than those injected withα subunit of Nav1.6 cRNAs alone (Fig. 2Ca,b), indicating that APP modulates α subunit of Nav1.6. In contrast, co-injection of cRNAs for the reversed sequence of APP and Nav1.6 α subunit, or for APP and α-subunit of Nav1.5, which is TTX-resistant subtype of sodium chnnel and predominantly expressed in hearts^17^, failed to enhance sodium currents (Fig. 2E), suggesting the specificity of modulation of Nav1.6 by APP. Consistent with the results obtained from hippocampal neurons of brain slices (Fig. 2A) and HEK293-Nav.16 cells (Fig. 2B), where α subunit of Nav1.6, β1 and β2 subunits are expressed endogenously, enhanced sodium currents by APP were still observed when coexpressed α subunit of Nav1.6 and β1 or/and β2 subunits (Fig. S2). In contrast, neither activation (Fig. S3Aa) nor inactivation curve of sodium currents (Fig. S3Ab) exhibited differences in between the oocytes co-injected with APP and Nav1.6 and those being injected with Nav1.6 alone. There was also no significant difference in recovery from inactivation between two groups (Fig. S3Ac). These observations suggest APP enhances sodium current density rather than modulating the dynamic capability of Nav1.6. Thefore, these results indicate that APP enhances sodium current density through modulation of α-subuit of Nav1.6.

### The VTPEER motif of APP increases sodium current expression in Xenopus oocytes expressing the Nav1.6 sodium channel α subunit

**Figure 3:**
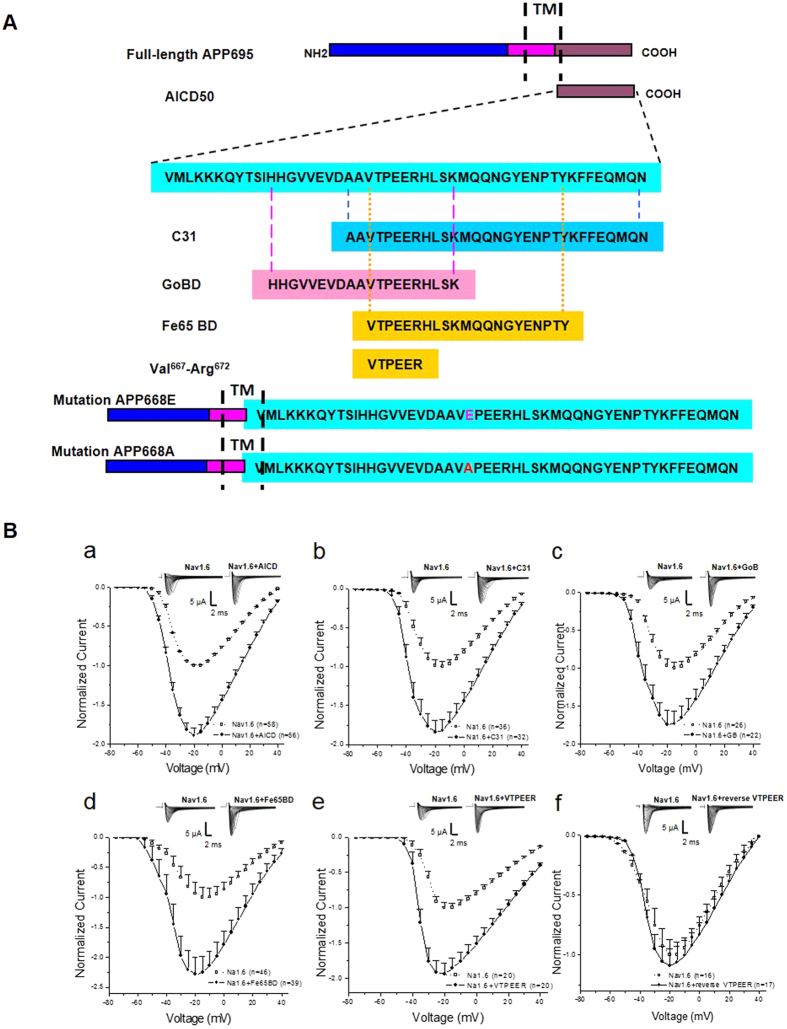
The effects of distinct APP intracellular domains on voltage-gated sodium channels. (**A**) Schematic representation of APP (full-length APP695), APP intracellular domain (AICD50), APP C31 domain (C31), APP Go protein binding domain (GoBD), Fe65 binding domain (Fe65BD) and VTPEER peptides (Val^667^-Arg^672^). (**B**) Normalized current-voltage relationships of currents in oocytes expressing Nav1.6 α subunit and together with APP intracellular domain (AICD50; (a); closed circles), APP C31 domain (C31; (b); closed circles), APP Go protein binding domain (GoBD; (c); closed circles), Fe65 binding domain (Fe65BD; (d); closed circles), VTPEER peptide (VTPEER; (e); closed circles) and the reversed VTPEER sequence (reverse VTPEER; (f); closed circles). The oocytes expressing Nav1.6 α subunit alone were recorded as control (a–f; open squares). Insert: Representative examples of sodium current responses elicited by depolarizing voltage steps.

APP is a type I transmembrane protein containing a relatively large extracellular domain and a short intracellular domain^18^. The cytoplasmic domain of APP binds to multiple proteins including Fe65^19^,^20^, Go^21^,^22^. We then determined which domain (s) in APP could be involved in enhancing sodium current expression in Xenopus oocytes expressing Nav1.6 sodium channel α subunit. We coexpressed APP intracellular domain (AICD^23^), Go protein-binding domain (GoBD; H^657^-K^676^ of APP-695), Fe65-binding domain^24^ (Fe65BD; V^666^-Y^686^, or C31 domain^25^ (C31; A^664^-N^695^) (Fig. 3A) with Nav1.6 α subunit in Xenopus laevis oocytes. Interestingly, all the intracellular domains, including AICD (Fig. 3Ba), C31 (Fig. 3Bb), GoBD (Fig. 3Bc) and Fe65BD (Fig. 3Bd) increased the peak sodium currents (Fig. 3Ba–d). In contrast, control injection of cRNAs for their reverse sequences failed to do so (not shown). Given that the VTPEER motif is a common overlapped peptide sequence in AICD, GoBD, Fe65BD, and C31 (Fig. 3A), we further coexpressed VTPEER peptide with Nav1.6 α subunit in Xenopus laevis oocytes. Notably, similar to full-length APP and the other intracellular domains, VTPEER increased the peak sodium currents (Fig. 3Be), while injection of control cRNA which encodes the reversed sequence of VTPEER failed to do so (Fig. 3Bf). All the intracellular domains had no significant effect on activation, inactivation or recovery from inactivation (Fig. S3B–E). These data demonstrate that APP modulates sodium currents of Nav1.6 through VTPEER motif. Considering the intracellular domain of APP (AICD), which is produced by proteolytic cleavage of APP, is elevated in the brains of AD patients^26^, theses data also raise a possibility that AICD, which lacks of the extracellular domain, similar to full-length APP, increases sodium currents of Nav1.6.

### APP enhances Nav1.6 currents through Go signalling

**Figure 4:**
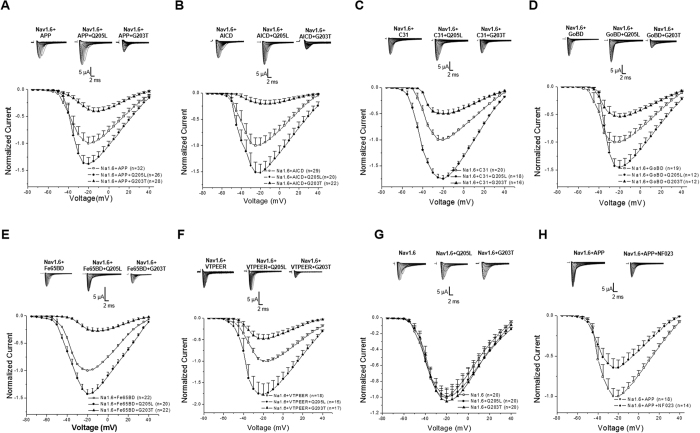
APP Increases Nav1.6 currents in a Go Protein-dependent manner. (**A–F**), Normalized current-voltage relationships of currents in oocytes expressing Nav1.6 α subunits and distinct APP intracellular domains alone (open squares; (**A**) Full-length APP; (**B**) AICD; (**C**) C31; (**D**) Go protein binding domain, GoBD; (**E**) Fe65 binding domain, FeBD; (**F**) VTPEER peptides together with a dominant active mutant of Go protein α subunit (Q205L, closed circles) or a dominant negative mutant of the Go protein α subunit (G203T; closed triangles). (**G**) Normalized current-voltage relationships of currents in oocytes expressing Nav1.6 α subunits alone (open squares) and together with a dominant active mutant of Go protein α subunit (Q205L, closed circles) or a dominant negative mutant of the Go protein α subunit (G203T; closed triangles). (**H**) Normalized current-voltage relationships of currents in oocytes expressing Nav1.6 α subunits and APP with (closed circles) or without (open squares) intracellular injection G protein inhibitor NF023. Insert: Representative examples of sodium current responses elicited by depolarizing voltage steps. Error bars represent mean ± S.E.

APP can act like a G protein-coupled receptor. An antibody, 22C11, to the extracellular domain of APP can act as a ligand mimetic to activate Go protein^21^. We thus investigated whether a Go protein-linked mechanism underlies the enhancement of Nav1.6 currents by APP. We co-expressed APP and Nav1.6 α subunit together with Go mutants in Xenopus laevis oocytes. Co-expression with a constitutively active mutant of Goα (Q205L)^27^ further increased the enhancement of Nav1.6 currents by APP, whereas a dominant negative mutant of Goα (G203T)^27^ suppressed the enhancement of Nav1.6 currents by APP (Fig. 4A). The similar changes of Nav1.6 currents modulated by Go mutants were found in distinct APP intracellular domains including AICD (Fig. 4B), C31 (Fig. 4C), Go binding domain (Fig. 4D), Fe65 binding domain (Fig. 4E) and VTPEER (Fig. 4F). However, in absence of APP, neither Q205L nor G203T showed any effects on sodium currents of Nav1.6 (Fig. 4G), indicating that Go signalling regulates Nav1.6 currents in an APP-dependent manner. Moreover, the APP-enhanced sodium currents was attenuated by intracellular injection NF203 (50 μM), one G protein inhibitor (Fig. 4H). These results demonstrate that APP-modulated sodium currents of Nav1.6 is regulated by Go signalling.

### Phosphorylation of APP Thr668 enhances sodium current expression

**Figure 5:**
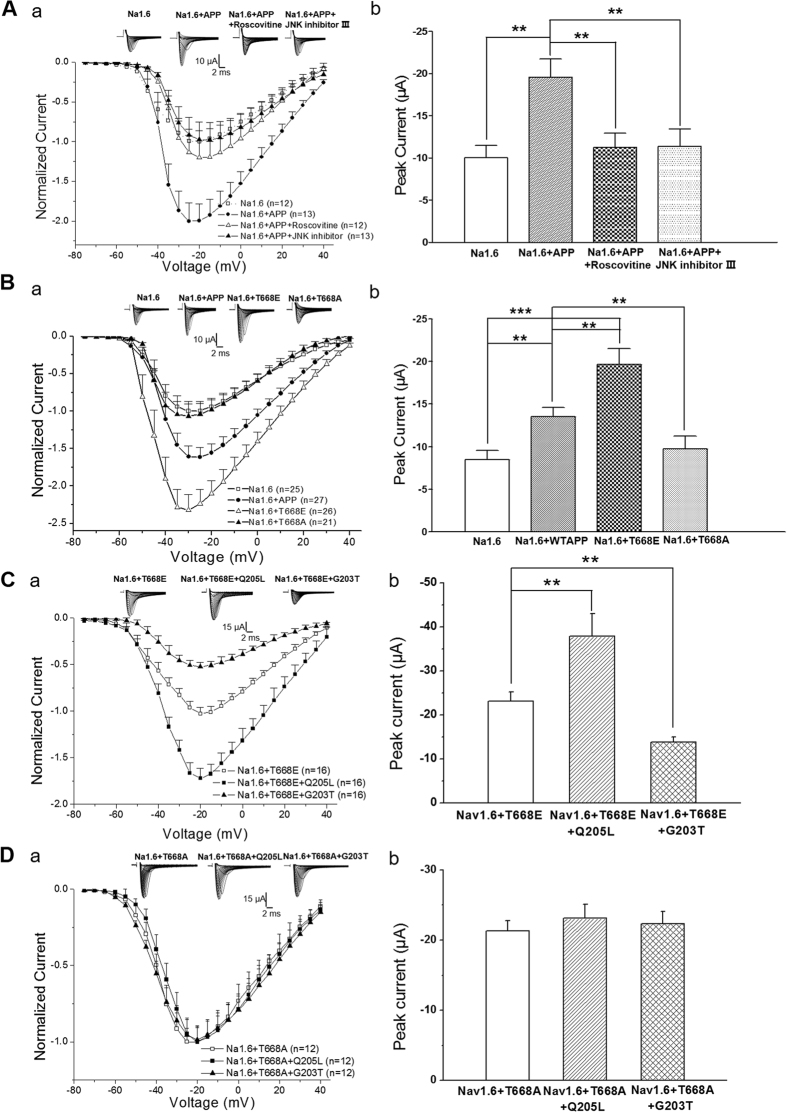
Thr-668 phosphorylation of APP enhances sodium current expression. (**A**) Normalized current-voltage relationships of currents in oocytes expressing Nav1.6 α subunits alone (a; open squares) and together with APP (a; closed circles), APP plus CDK5 inhibitor (a; Roscovitine 10 μM; open triangles) and APP plus JNK inhibitor (a; JNK inhibitor III 7 μg/ml, closed triangles). The mean peak sodium current amplitude in oocytes injected with Nav1.6 and APP and applied with the indicated inhibitors (b). (**B**) Normalized current-voltage relationships of currents in oocytes expressing Nav1.6 α subunits alone (a; open squares) and together with wild-type APP (a; closed circles), APP T668E (a; open triangles), and APP T668A (a; closed triangles). The mean peak sodium current amplitude in oocytes injected with Nav1.6 and the indicated APP or APP mutant cRNAs (**b**). (**C**) Normalized current-voltage relationships of currents in oocytes expressing Nav1.6 α subunits with APP T668E alone (open squares) and together with a dominant active mutant of Go protein α subunit (Q205L, closed squares) or a dominant negative mutant of the Go protein α subunit (G203T; closed triangles) (a). The mean peak sodium current amplitude in oocytes injected with Nav1.6 and T668E and the Go mutant cRNAs (b). (**D**) Normalized current-voltage relationships of currents in oocytes expressing Nav1.6 α subunits with APP T668A alone (open squares) and together with a dominant active mutant of Go protein α subunit (Q205L, closed squares) or a dominant negative mutant of the Go protein α subunit (G203T; closed triangles) (a). The mean peak sodium current amplitude in oocytes injected with Nav1.6 and T668A and the Go mutant cRNAs (b). Insert: Representative examples of sodium current responses elicited by depolarizing voltage steps. One-way ANOVA. **p < 0.01; Error bars represent mean ± S.E.

VTPEER contains a tyrosine residue (Thr668), which is a neuronal specific phosphorylation site of APP^28^. Given that phosphorylation has been shown to regulate binding of a number of proteins to APP intracellular domain, we investigated whether phosphorylation of APP at Thr668 might be involved in the functional interaction between APP and Nav1.6. In the brain, phosphorylation of APP at Thr668 is mediated by neuronal CDK5^29^, GSK-3β^30^, c-Jun N-terminal kinase (JNK) 3^31^. JNK has been implicated as a downstream signal transducer of Go in APP-mediated cell death^32^. We thus applied inhibitors for JNK and CDK5 when recording sodium currents in Xenopus laevis oocytes coexpressing APP with Nav1.6 α subunit. Interestingly, the enhancement of sodium currents by APP were blocked by application of JNK inhibitor (7 μg/ml JNK inhibitor III) or CDK5 inhibitor (10 μM Roscovitine) for 5 minutes (Fig. 5Aa,b), suggesting that phosphorylation of APP (Thr668) is involved in modulation of Nav1.6. To confirm this idea, two APP point mutants that have a Glu or Ala substitution at Thr668, respectively, were coexpressed with Nav1.6 α subunit in Xenopus laevis oocytes. The Glu substitution (T668E), which mimicks the negative charges of phosphorylation^33^, increased sodium current expression. In contrast, the Ala substitution (T668A), which abolishes phosphorylation of APP^33^, failed to change sodium current expression (Fig. 5Ba,b). These results indicate that phosphorylation of APP at Thr668 site is required for modulation of sodium currents of Nav1.6 by APP. Moreover, co-expression with Go Q205L, a constitutively active Goα, further increased the enhancement of sodium currents induced by T668E, whereas co-expression of Go G203T, a dominant negative Goα, decreased the enhancement of sodium currents (Fig. 5Ca,b). In contrast, neither Go Q205L nor Go G203T exhibited any effect on sodium currents of Nav1.6 when they were co-expressed with T668A (Fig. 5Da,b), indicating that phosphorylation of APP at T668 sites is required for modulation of Nav1.6 by Go signalling. Together, these results indicate that APP modulates α-subunit of Nav1.6 in a phosphorylation (Thr668)-dependent manner, which can be regulated by activity of Goα.

### APP regulates cell surface expression of Nav1.6 dependent on phosphorylation of Thr668

**Figure 6:**
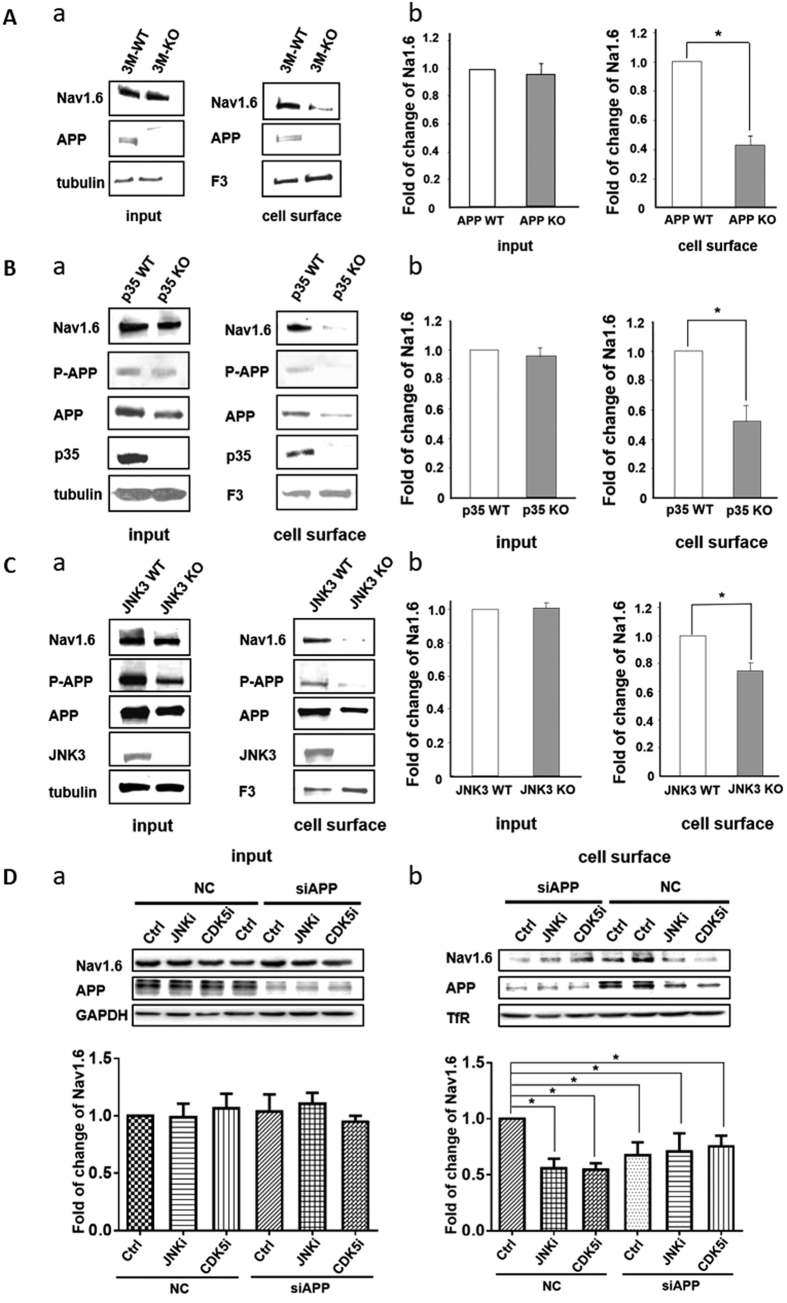
The cell surface expression of Nav1.6 sodium channels in APP, p35 and JNK3 knockout mice and Hek 293-Na1.6 cells for virous treatments. Analysis of cell surface expression of Nav1.6 protein in the white matter of spinal cord isolated from mutant mice and Hek 293-Na1.6 cells. Total proteins and cell surface proteins, biotinylated and pull-down by NeutrAvidin Gel, were subjected to Western blots (n = 3). (**A–C**) Total proteins and cell surface proteins isolated from the ventral white matter of spinal cords of APP KO (Aa), p35 KO (Ba), JNK3 KO (Ca) and their littermate WT mice were blotted using antibodies against Nav1.6, APP, phosphorylated APP at T668 (p-APP), F3, p35, JNK3 and γ-tubulin. Quantification of the level of protein expression in input and cell surface. The levels of Nav1.6 in the WT spinal cords were normalized to 1.0, the relative levels of Nav1.6 in KO mice were quantified (b). (**D**) HEK293-Nav1.6 cells were transfected APP siRNA or a scrambled siRNA (NC) and then treated with JNK inhibitor III (JNKi, 10 μg/ml), CDK5 inhibitor (CDK5i, 20 μM Roscovitine) or vehicle/DMSO (Control, Ctrl). Western blot analysis of the cell surface expression or the total protein of Nav1.6 using antibodies against Nav1.6 and APP. GAPDH and (transferrin receptor, TfR) were loaded as control. The levels of Nav1.6 in DMSO-treated NC-transfected cells were normalilzed to 1.0. The relative levels of Nav1.6 in other groups of the cells as indicated were quantified. Student t test. *, *p* < 0.05; **, *p* < 0.01. Error bars represent mean ± S.E.

APP enhances sodium current density without affecting the activation, inactivation and recovery from inactivation, suggesting a role of APP in regulation of trafficking of Nav1.6 to the cell surface. APP colocalizes with Nav1.6 at NORs of myelinated axons^1^, we thus investigated expression of Nav1.6 on the cell surface in the white matter of spinal cord of APP KO mice, where myelinated axons are enriched. Both total and cell surface proteins were prepared and subjected to Western blotting using antibodies against Nav1.6 sodium channel α subunits and F3/contactin, a nodal marker molecule in the CNS^34^. There was no discernable difference in total Nav1.6 sodium channel expression in the white matters of APP KO and WT spina cords (Fig. 6Aa,b), suggesting that APP does not change Nav1.6 expression. However, the levels of Nav1.6 sodium channel on the cell surface was decreased in the white matters of APP KO spinal cords compared to that in WT spinal cords (Fig. 6Aa,c). In the adult, Nav1.6 is the predominant α subunit of sodium channel locating at the mature NORs, whereas Nav1.2 is mainly located at the immature NORs in the developing nervous system^7^. Consistent with the results that APP does not modulate sodium currents of Nav1.2 (Fig. S1), the cell surface expression of Nav1.2 was not changed upon transfection of APP in HEK293-Nav1.2 cells (Fig. S4). As the protein samples were isolated from the white matter of adult spinal cord, these results imply that APP may play a role in modulating the cell surface expression, but not the protein expression, of Nav1.6 sodium channels, at the CNS NORs.

Given that APP modulates sodium currents of Nav1.6 depending on phosphorylation of APP (Thr668), we investigated whether phosphorylation of APP (Thr668) regulates cell surface expression of Nav1.6 in the myelinated axons. The white matters of spinal cords from p35 KO and JNK3 KO mice were isolated to subject to analysis of cell surface protein. p35 is a noncyclin activator that activates CDK5^35^, which phosphorylates APP at Thr668. Consistent with previous reports^9^, the levels of phosphorylated APP (Thr668) decreased in the white matters of p35 KO or JNK3 KO spinal cords (Fig. 6Ba,Ca). Notably, similar to the results obtained from APP KO mice, the levels of Nav1.6 α subunit on the cell surface decreased in the white matters of p35 and JNK3 KO spinal cords, compared to those in WT spinal cords (Fig. 6B,C). To further confirm the reduction in cell surface expression of Nav1.6 observed in p35 and JNK3 KO is due to reduced phosphorylation of APP (Thr668), HEK293-Nav1.6 cells were transfected with siRNA targeting to APP, which down-regulated endogenous expression of APP, and treated with JNK inhibitor (10 μg/ml JNK inhibitor III) or CDK5 inhibitor (20 μM Roscovitine). In HEK293-Nav1.6 cells which were transfected with a scrambled siRNA (NC), cell surface expression of Nav1.6 were reduced by treatment with CDK5 and JNK inhibitors (Fig. 6D). In contrast, neither CDK5 inhibitor nor JNK inhibitor decreased cell surface levels of Nav1.6 in APP siRNA-transfeced HEK293-Nav1.6 cells (Fig. 6D), indicating that inhibition of CDK5 or JNK reduces cell surface expression of Nav1.6 through APP. The total protein levels of Nav1.6 exhibited no differences in between APP siRNA- and NC-transfected cells with or without treatment of CDK5 inhibitor or JNK inhibitor (Fig. 6D). Thus, these results demonstrate that phosphorylation of APP (Thr668) is essential for modulation of the cell surface insertion of Nav1.6 sodium channels in the myelinated axons in CNS.

**Figure 7:**
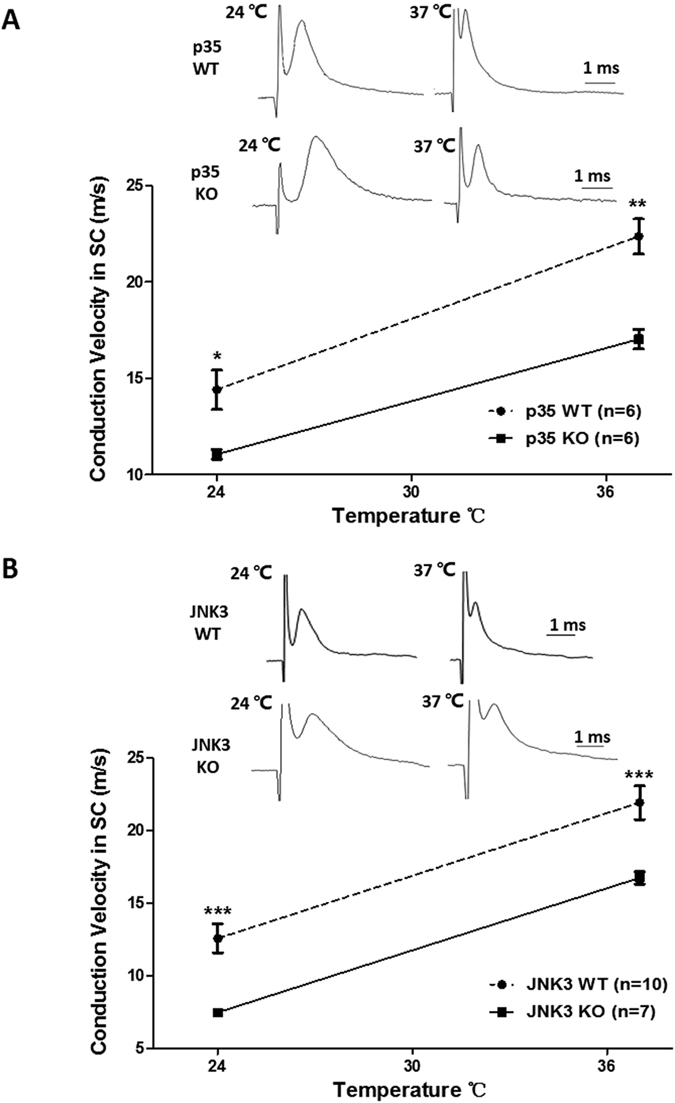
Conduction velocities of compound action potentials in the spinal cords of p35 and JNK3 knockout mice. Determination of conduction velocities of CAPs in p35 (**A**) and JNK3 (**B**) KO and WT mice. Representative CAPs from spinal cords of p35 (p35 KO; n = 6) and JNK3 (JNK3 KO; n = 7) KO and their wild-type mice (n = 6 for P35 WT; n = 10 for JNK3 WT). Representative CAPs recorded at 24 °C and 37 °C from spinal cords of APP KO and WT mice (upper panel). The conduction velocity measured in WT mouse spinal cord (open bars) was consistently higher than that seen in APP KO mice (closed bars) at both temperatures measured (lower panel). N numbers are indicated inside the bars. Student’s t-test, *p < 0.05; **p < 0.01. Error bars represent mean ± S.E.

Consistent with the results that P35 and JNK3 KO mice exhibited decreased cell surface expression of Nav1.6 through phosphorylation of APP, which positively modulates sodium currents of Nav1.6, the CAPs in the spinal cords of p35 and JNK3 KO mice decreased in comparison to their littermate WT mice at both 24 °C and 37 °C (Fig. 7B). In contrast, there was no significant difference in the conduction velocities between p35 and JNK3 WT mice (not shown). Thus, these results reveal that APP increases sodium currents through enhancing cell surface expression of Nav1.6 in a phosphorylation (APP Thr668)-dependent manner.

## Discussion

AD patients have an increased incidence of epileptic seizures, and the incidence is even higher in patients with early-onset AD who overexpress human APP^5^. Hyperexcitability is also detected in the brains of various AD transgenic mice^36^. Such aberrant increases in network excitability and compensatory inhibitory mechanisms in the hippocampus may contribute to the cognitive deficits in AD^37^,^38^,^39^. However, the mechanisms underlying hyperexcitability detected in AD brains are not fully understood. In the present study, we show a functional interaction between APP and Nav1.6, a key determinant of neuronal excitability^40^. We describe that APP enhances sodium currents of α-subunit of Nav1.6 in a phosphorylation (Thr668)-dependent manner. Our findings are also consistent with the facts that both APP and Nav1.6 are involved in the pathogenesis of epilepsy^40^,^41^,^42^.

It has been reported that BACE1 and γ-secretase could cleave β2-subunit of Nav1.1 sequentially and then release the intracellular domain of β2-subunit from the membrane, which in turn regulates transcription of Nav1.1^43^. The sequential cleavage of APP by BACE1 and γ-secretase generates Aβ and AICD. However, it seems unlikely that the modulation of sodium current density by APP that we have observed in the present study is due to dysregulation of BACE1 and γ-secretase, since AICD, even VTPEER, which obviously are not substrates of BACE1 and γ-secretase, enhances sodium current density of Nav1.6 α-subunit in Xenopus oocytes. Due to endogenous expression of β1 and β2 subunits in HEK293 cells^13^,^14^, the previous report cannot exclude a possibility that a functional interaction of APP and β1 or β2 subunit^9^. In the present study, we have further observed that APP enhances sodium current density in the oocytes co-injected cRNA for APP and α-subunit of Nav1.6, where there are few levels of β1 and β2 subunits. Thus, the present study further identify that APP modulates α-subunit of Nav1.6, which is independen on β1 or β2 subunit.

AICD contains a Go protein-binding domain (His657-Lys676). Moreover, an antibody, 22C11, to the extracellular domain of APP can act as a ligand mimetic to activate Go protein through His657-Lys676, demonstrating that APP can act like a G protein-coupled receptor^21^, although other study reported a controdictary observation that 22C11 inhibits Go protein^22^. Our results demonstrate that the APP-induced increased Nav1.6 currents is mediated by the His657-Lys676 peptide, Go protein binding domain in the intracellular domain of APP695, and appears to involve the heterotrimeric G protein Go. This Go protein binding domain is known to selectively activate the heterotrimeric G protein Go with which it complexes. This is consistent with the observations by Liu *et al*., that APP activates Go protein, which in turn increases phophorylation of APP (Thr668) through activating JNK. Upon phosphorylation, the binding of APP to Nav1.6 is enhanced^9^. In the present study, we further show that VTPEER, a short sequence within Go binding domain of APP, modulates sodium current density in a Go-dependent way, which is consistent with the previous study that APP binds and activates Go signaling through His657-lys676 domain^22^. Thus, we propose that APP activates Go through binding to VTPEER. However, the fact that the peptides, which encode the intracellular domain of APP containing VTPEER, modulate Nav1.6 also raises another point that full-length APP is not required for modulating Nav1.6. Considering that elevated levels of AICD are observed in the brains of AD patients^44^, our results implicate that in addition to full-length APP, AICD, is also an important contributor to the hyperexcitability observed in the brains of AD patients. Moreover, similar to full-length APP, the enhanced sodium currents of Nav1.6 by the peptides encoding APP intracellular domain containing VTPEER including AICD are increased or decreased by constitutively active Goα and dominant negative Goα, respectively. However, it is worth noting that AICD is lack of the extracellular domain, which is required in activation of Go signaling^21^. Thus, in contrast to full-length APP which activates Go signaling as a receptor, AICD modulates Nav.16 currents in a disctinct way.

**Figure 8:**
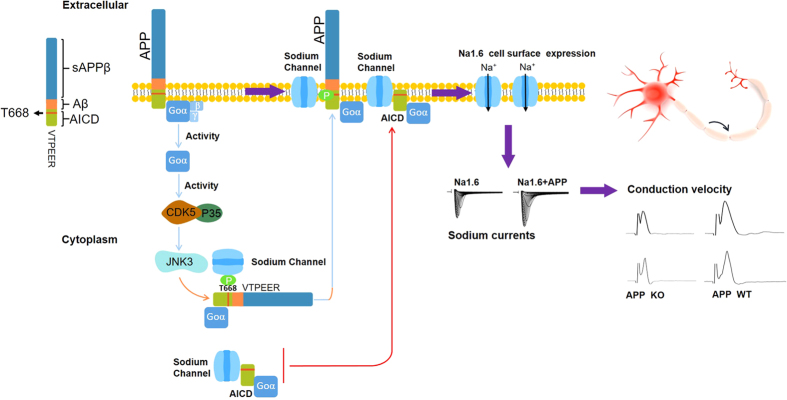
Schematic model for regulation of Nav1.6 by APP. APP through VTPEER activates Go protein, which enhances phosphorylation of APP (Thr668) by activating of JNK3. Phosphorylated APP (Thr668) binds to Nav1.6 and promotes cell surface expression of Nav1.6. In addition, activation of Goα further enhances the functional interaction between phosphorylated APP (Thr668) and Nav1.6. AICD, after cleavage from full-length APP, upon being phosphylated (Thr668), increases cell surface expression of Nav1.6 as well, which is also mediated by activity of Goα. Phosphorylated APP/AICD (Thr668) mediates cell surface expression of Nav1.6 may through promoting Nav1.6 insert into the cell surface or maintaining Nav1.6 on the cell surface.

Neuron-specific phosphorylation of APP695 at Thr668, within the Go protein-binding domain of APP, is important for neuronal functions of APP, which can induce a conformational switch in APP and alter its binding specificity and affinity to cytosolic partners^45^. For instance, Thr668 phosphorylation plays a role in APP metabolism by facilitating the BACE cleavage of APP to increase Aβ generation^46^ and is required for AICD binding to Fe65^33^ and translocation into the nucleus^47^. Consistently, our group have recently shown that phosphorylation of APP695 at Thr668 enhances its interaction with Nav1.6^9^. Thus, we propose that activation of Go by APP enhances activity of JNK, which in turn, increases phosphorylation of APP695 at Thr668. The latter increases cell surface expression of Nav1.6 through enhancing its binding to Nav1.6^9^. However, in the present study, we find that phosphorylated APP695 at Thr668 modulates Nav1.6 currents through a way more complicated than that. Sodium current density of Nav1.6 is enhanced by a constitutively active Goα, whereas it is suppressed by dominant negative Goα, in presence of APPT668E. In contrast, neither constitutively active Goα nor dominant negative Goα have any effect on sodium current density of Nav1.6 in presence of APPT668A which abolishes the phosphorylation of APP at Thr668, or in absence of APP/AICD. These results suggested an additional mechanisms underlying that Go protein in modulating sodium current density of Nav1.6, rather than through enhancing phosphorylation of APP at Thr668 by activating JNK. Because if Go modulates Nav1.6 currents through enhancing phosphorylation of APP at Thr668, when APPT668E is coexpressed, which would not be further phosphorylated, neither constitutively active Go nor dominant negative Go would have any effect on Nav1.6 currents. Therefore, we propose Go protein may play dual roles in modulation of Nav1.6 currents. Upon activation by full-length APP, it enhances phosphorylation of APP at Thr668 by activation of JNK. In the other hand, active Go signaling increases the functional interaction between phosphorylated APP/AICD (Thr668) and Nav1.6, further enhancing the capability of phosphorylated APP/AICD (Thr668) in modulating Nav1.6 (Fig. 8). This also can explain why modulation of Nav1.6 currents by VTPEER, Fe65 binding domain, AICD, which lack of extracellular domain, is mediated by Go activity. However, it remains further investigated on how Go activity modulates functional interaction between phosphorylated APP/AICD (Thr668) and Nav1.6.

Therefore, the present study is complementary to our preious report that APP positively regulates sodium currents of Nav1.6 through activating Go protein, which enhances phosphorylation of APP (Thr668) via activation of JNK. Upon phosphorylated, APP enhances its binding to Nav1.6 and promoteing cell surface expression of Nav1.6^9^. The present study confirms our previous reports with different experimental systems and further identify that i) APP could modulate α-subunit of Nav1.6 independent on β1/β2 subunit; ii) APP interacts α-subunit of Nav1.6 through VTPEER; iii) Similar to full-length APP, APP intracellular domain containing VTPEER positively regulates sodium currents of Nav1.6, that is mediated by Go activity; iv) Go protein modulates Nav1.6 currents in a phosphorylated APP/AICD (Thr668)-dependent manner, which plays dual roles in APP/AICD-medaited Nav1.6 currents: *a*) Upon being activated by full-length APP, Go enhances phosphorylation of APP (Thr668) through activation of JNK; *b*) Go protein may enhance the functional interaction of phosphorylated APP/AICD (Thr668) with Nav1.6.

## Conclusions

The findings of the current study support APP/AICD modulates Nav1.6 sodium channels through a Go-coupled JNK pathway, which is dependent on phosphorylation of APP at Thr668.

## Materials and Methods

### Antibodies and inhibitors

The rabbit polyclonal antibody against APP was as described previously. Rabbit polyclonal anti-Nav1.6 (Alomone Laboratories), c-Jun N terminal protein kinase (JNK) (J4500; Sigma), p35 (cell signalling), F3 (Mellita), actin (abcam), TfR (Boster), GADPH (Abcam) and γ-tubulin (GTU88; Sigma) were obtained from the respective commercial sources. NF203 (Sigma), JNK inhibitor III (Calbiochem) and roscovitine (Calbiochem) were purchased.

### Mutant mice

The APP KO, p35 KO and JNK3 KO mice have been previously described. All experiments involving mice were approved and performed in accordance with the regulations of the Institutional Animal Care and Use Committee (IACUC) of Singapore General Hospital and the animal studies committees of the Dalian Medical University (Ethics committee approval permit No. L2013011). The animals were maintained under conditions of 12-h light/dark cycle with food and water available ad libitum. The experiments involving Xenopus laevis oocytes were approved by the IACUC of the National University of Singapore.

### Cell Surface biotinylation

Cell surface biotinylation was performed according to a protocol previously described^43^. Brifely, ventral spinal cords (containing mostly white matter) were isolated and incubated with 0.5 mg/ml sulpho-NHS–Biotin (Pierce) in ASCF bubbled with 95% O_2_ and 5% on ice for 30–40 min. The reaction was stopped by quench buffer (Pierce). The tissues were then extracted in a lysis buffer containing 10 mM Tris-HCl at pH 9.0, 150 mM NaCl, 0.5% triton x-100, 1% sodium dexycholate, 0.5% SDS, 2 mM EDTA and protease inhibitors. Biotinylated proteins were captured by Streptavidin beads (Pierce) at 4 °C overnight.

### Western blot analysis

To prepare whole extracts, frozen tissues (spinal cords and brains) were homogenized in a lysis buffer (50 mM Tris-HCl pH 7.5, 5 mM EDTA, 1% Triton X-100, and protease inhibitors (Roche Diagnostics). After ultracentrifugation (150,000 g, 4 °C, 45 min), the supernatants were collected and stored at −80 °C for future use. All samples were subjected to Bradford protein assay.

Equal amounts of protein were separated on acrylamide gels and transferred onto nitrocellulose membranes. Western blotting was performed under standard conditions, applying rabbit polyclonal antibodies against APP (1:1000), and Nav1.6 (1:200) mouse monoclonal antibodies against γ-tubulin (1:1000). The latter protein was used for loading normalization. Either anti-mouse or anti-rabbit peroxidase-conjugated secondary antibodies were applied at 1:10,000 and blots were visualized with an ECL^TM^ detection kit (Amersham).

### Conduction velocity recording

Electrophysiology for spinal cord: Conduction properties of myelinated axons were examined in acutely isolated spinal cords of C57BL/6 mice, using a double grease-gap technique. The spinal cord was placed in a Plexiglas chamber, with the central channel (15 mm wide) superfused by flowing, oxygenated Krebs’ solution and the two ends isolated by grease gaps in chambers containing isotonic (120 mM) potassium chloride solution. Silver/silver chloride electrodes were used to record the potential across one gap and to stimulate the spinal cord across the other. This provided a stable recording and stimulating arrangement with a fixed conduction distance of 15 mm for the spinal cord. The animals were anesthetized, and then decapitated and the spinal cord extracted by rapid laminectomy and washed in oxygenated Krebs’ solution (NaCl 119 mM, KCl 2.5 mM, NaH_2_PO_4_ 1 mM, MgSO_4_ 1.3 mM, CaCl_2_ 2.5 mM, D-glucose 11 mM, NaHCO_3_ 26.2 mM, equilibrated with 95% O_2_, 5%CO_2_). The cord was incubated in Krebs’ solution at room temperature for at least 1 hr, and then mounted in a recording chamber. The temperature of chamber was raised from room temperature at 24 °C to 37 °C, and a series of recordings were made to stimulation that was adjusted to 10% above the level that elicited a maximum response. The data were digitized at 20 kHz for subsequent analysis.

Electrophysiology for sciatic nerve: Sciatic nerves were dissected immediately after animals were killed and placed in oxygenated Krebs’ solution. Nerves were then transferred to a recording chamber that was continuously perfused with oxygenated Krebs’ solution. Stimulation was delivered onto one end of nerve with a concentric bipolar electrode (Frederick Haer Co.) and the other end of the nerve was drawn into a suction electrode for recording CAPs^10^.

### Hippocampal slice preparations and electrophysiological patch clamp recording

The animals from anesthetized 21–35 day-old APP Knock Out and Wide Type littermate mice were anesthetized with isofluorane in agreement with the guidelines of Institutional Animal Care and Use Committee. The brains were removed and placed in anoxygenated (95% O_2_/5% CO_2_) artificial cerebrospinal fluid (ACSF) at 4 °C, containing (in mM) (126 NaCl, 2.5 KCl, 1 NaH_2_PO_4_, 2.5 CaCl_2_, 1.5 MgSO_4_, 26 NaHCO_3_ and 10 glucose). The osmolarity of the ACSF was adjusted to 300 mOsm and the pH was adjusted to 7.2. Acute brain slices (300 μm thick) were obtained using a vibrating blade microtome (Lica VT1200, Lica instruments, Nussloch, Germany). The slices were gently transferred to a submersion holding chamber containing ACSF bubbled with 95% O_2_ and 5% CO_2_. Slices were equilibrated at room temperature for at least 1 h before being transferred to a submersion-recording chamber, which was continually perfused with oxygenated ACSF at a rate of 1–2 ml/min at room temperature.

Whole-cell recordings were obtained from CA1 pyramidal neurons of mice hippocampus or HEK 293 cells stably expressing Nav1.6 cells with borosilicate pipettes that were pulled from borosilicate glasses (Boralex) with a Flaming Brown micropipette puller (P-97, Sutter Instrument Co., CA, USA). The pipettes resistances of 2–6 MΩ were filled with a solution that contained (mM): 140 CsCl, 2 MgCl_2_, 10 EGTA, 2 Na_2_ATP 20 HEPES, pH 7.3 and was adjusted to 300 mOsm/L with Sucrose. CdCl_2_ in bath solution was used to block Ca^2+^ currents. Cells were clamped using MultiClamp 700B amplifier (Molecular Devices Corp., Sunnyvale, CA, USA) in conjunction with a Digidata 1322 A interface (Axon Instruments, Union City, CA, USA) at a holding potential of −70 mV. Currents were recorded and analyzed using pCLAMP 9.2 software (Molecular Devices Corp.). Data were analyzed by one-way ANOVA, followed by Student’s t-test for paired group (two tailed) with *p* < 0.05 indicating a significant difference.

Voltage command protocols were as follows. Standard current–voltage (I–V) families were obtained using 50 msec pulses from a holding potential of −70 mV to a range of potentials (−60 to 60 mV) in 10-mV increments. The peak value at each potential was plotted to form I–V curves. Activation curves were fitted with the following Boltzmann distribution equation: G_Na_/G_Na_,_max_ = 1/[1 + exp(V_1/2_ - Vm)/k], where G_Na_ is the voltage-dependent sodium conductance, G_Na,max_ is the maximal sodium conductance, V_1/2_ is the potential at which activation is half-maximal, V_m_ is the membrane potential, and k is the slope. Inactivation protocols consisted of a series of pre-pulses (−100 to 10 mV) lasting 100 ms from the holding potential of −70 mV, followed by a 100 ms depolarization to −10 mV. The normalized curves were fitted using a Boltzmann distribution equation: I_Na_/I_Na_,max = 1/[1 + exp((Vm-V_1/2_)/k)], where I_Na, max_ is the peak sodium current elicited after the most hyperpolarized prepulse, V_m_ is the preconditioning pulse potential, V_1/2_ is the half-maximal sodium current, and k is the slope factor. For recovery from inactivation experiments, two 40 ms stimuli were given to −10 mV from the holding potential of −110 mV, with a variable recovery time period at −110 mV, in the range of 1 to 400 ms. The peak amplitude of the second sodium current response divided by the response at the maximal interval was then plotted as a function of the interpulse interval. Curves were fitted with a double rising exponential function.

### Cell Culture and Transfection

HEK 293 cells stably expressing Nav1.6 were obtained from Dr. J. J. Clare^12^ and grown in DMEM supplemented with 10% (v/v) FBS and 400 μg/ml G418 (Invitrogen). HEK293 Nav1.6 cells were transfected with full-length APP plasmids using Effectene transfection reagent (Qiagen) or with siRNA using Lipofectamine RNAiMAX transfection reagent (Invitrogen) according to the instructions of the manufacturer. Two days after transfection, the cells were used for experiment.

### Plasmid preparation and cRNA transcription

The pLCT1-Scn8a plasmid containing mouse cDNA of Nav1.6 α subunit of sodium channels was provided by Dr. Alan L. Goldin (University of California, Irvine). The plasmid containing cDNA of Nav1.5 α subunit of sodium channels was provided by Dr A.L. George (Vanderbilt University, Nashville). The plasmid containing the human APP-695 cDNA was provided by Dr. Carsten Schmidt (University of Hamburg). Moreover, we constructed a vector pD4GL, a modified version of pcDNA4A (Invitrogen), which uses the Xenopus β-globin untranslated regions to boost expression in oocytes. The Xenopus β-globin 5′ and 3′ untranslated regions were subcloned into the pcDNA4A vector. The AICD domain and G protein Go binding domain of human APP were inserted between these two regions. To facilitate translational recognition by ribosomes, a Kozak sequence was added to around the start codon (GCCACCATGG) for oocyte expression. The AICD domain (forward primer: 5′-TTTGGTGAAGCTTGCCACCATGGTGATGCTGAAGAAGAAACAGTAC-3′; reverse primer: 5′-GGCGGCGGCCGCCTAGTTCTGCATCTGCTCAAAGA-3) and the His657- Lys676 G protein Go binding domain (forward primer: 5′-GCGCAAGCTTGCCACCATGC ATCATGGTGTGGTGGAGGTTG-3′; reverse primer: 5′- GGCGGCGGCCGCCTACTTGGACAGGTGGCGCTCCTC-3′) and C31 domain (forward primer: 5′-GCGCAAGCTTGCCACCATGGCCGCTGTCACCCCAGAGGAG-3′; reverse primer: 5′-GGCGGCGGCCGCCTAGTTCTGCATCTGCTCAAAGA-3′); Fe65 domain (forward primer:5′-GCGCAAGCTTGCCACCATGGTCACCCCAGAGGAGCGCCAC-3′; reverse primer: 5′-GGCGGCGGCCGCCTACTTGTAGGTTGGATTTTCGT-3′); VTPEER domain (forward primer: 5′-AGCTTGCCACCATGGTCACCCCAGAGGAGCGCTAGGC -3′; reverse primer: 5′-GGCCGCCTAGCGCTCCTCTGGGGTGACCATGGTGGCA-3) were obtained from the full length human APP-695. The sequences of APP siRNA were as follows: 5& -CCAACCAACCAGUGACCAU[dT][dT] and 5& -AUGGUCACUGGUUGGUUGG [dT][dT], synthesized by Sigma. Plasmids were purified using QIAGEN Plasmid Maxi Kit (QIAGEN). All these purified constructs were linearized, and cRNAs were synthesized *in vitro* using the mMESSAGE mMACHINETM T7 cRNA synthesis kit (Ambion). The concentration of cRNA was determined from OD (260 nm).

### Expression and Electrophysiological Recording in Oocytes

Stage V oocytes were surgically removed from mature female Xenopus laevis (Xenopus Express) anaesthetized with 1 g/L tricane methanesulphonate (Sigma). Segments of ovarian lobe were removed by a small abdominal incision. The follicular layer was removed by digestion for 90 min with 2 mg/ml collagenase type V (Sigma) in Ca^2+^ -free Barth’s solution containing (mmol/L) NaCl 88, KCl 1, NaHCO_3_ 2.4, MgSO_4_ 0.82, Tris/HCl 5; pH 7.4 with NaOH; supplemented with 50 μg/ml gentamicin sulphonate). The oocytes were placed in 96-well plates and incubated at 19 °C in Barth’s solution overnight. On the following day oocytes were injected (Roboocyte) with cRNAs. RNA was dissolved in 1 mM Tris-HCl, pH 7.5. The molar ratio of the α subunit to APP was 1:1. The oocytes were then incubated at 19 °C. Membrane currents were recorded from oocytes with the two-microelectrode voltage-clamp technique at room temperature (19 °C to 21 °C) 2 to 3 days after injection of cRNA. Current injecting and potential measuring electrodes had resistances of 0.1 to 0.7 MΩ when filled with 3 mol/L KCl. Data acquisition was performed with a GeneClamp 500B two-electrode voltage clamp amplifier connected to a Digidata 1322 A interface and controlled with pClamp 9.2 software (Axon Instruments). Oocytes were perfused with ND96 solution containing (mmol/L) NaCl 96, KCl 2, CaCl_2_ 1.8, MgCl_2_ 1, and HEPES 5 (adjusted to pH 7.4 with NaOH). Oocytes were kept in current-clamp mode for at least 2 minutes before switching to voltage-clamp mode. All recordings were obtained after stable baseline and ionic current levels were achieved. Populations of oocytes were sampled from 4 frogs for each expression combination. Data was sampled at 50 kHz after filtering at 5 kHz with a 4-pole Bessel filter.

Oocytes were held at −100 mV and 45 ms steps to command potentials between −75 mV and 40 mV were made in 5 mV increments to assess the current-voltage (I-V) relationship. To plot the I-V relationship, the currents were normalized to the mean peak current in the absence of APP for each frog For statistical analysis, the peak current in each oocyte was determined. Activation was assessed by calculating conductance using the equation G = I/(V-E_Na_) (where I is the peak current amplitude at command potential V and E_Na_ is the equilibrium potential for sodium under our experimental conditions) and fitting the data to a Boltzmann distribution function: G_Na_/G_Na,max_ = 1/[1 + exp(V_1/2_-V_m_)/k], where G_Na_ is the voltage-dependent sodium conductance, G_Na,max_ is the maximal sodium conductance, V_1/2_ is the potential at which activation is half-maximal, V_m_ is the membrane potential, and k is the slope. Inactivation was assessed with a series of pre-pulses (−120 to 10 mV) lasting 500 ms from the holding potential of −100 mV, followed by a 25 ms depolarization to −10 mV. The normalized curves were fitted using a Boltzmann distribution equation (I_Na_/I_Na,max_ = 1/[1 + exp(V_m_ -V_1/2_)/k]), where I_Na, max_ is the peak sodium current elicited after the most hyperpolarized prepulse, V_m_ is the preconditioning pulse potential, V_1/2_ is the voltage at which the magnitude is half-maximum, and k is the slope factor. Current recovery from inactivation was assessed with two 50 ms pulses to −10 mV from the holding potential of −100 mV, with a variable recovery time period at −100 mV, in the range of 1 to 30 ms. Curves were fitted with a single rising exponential function. 100 μM Roscovitine (Calbiochem), a CDK5 inhibitor and 700 μg/ml JNK inhibitor III (Calbiochem) were applied. All data are expressed as mean ± SEM. Student’s t test was used to test for statistical significance of effects on peak current amplitude and V_1/2_. Two-way ANOVA for effects of APP and recovery time was used to analyze the data on recovery from inactivation. A value of *p* < 0.05 was considered significant.

## Additional Information

**How to cite this article**: Li, S. *et al*. Amyloid precursor protein modulates Nav1.6 sodium channel currents through a Go-coupled JNK pathway. *Sci. Rep.* **6**, 39320; doi: 10.1038/srep39320 (2016).

**Publisher's note:** Springer Nature remains neutral with regard to jurisdictional claims in published maps and institutional affiliations.

## Supplementary Material

Supplementary Information
