# Supplementary material for: Amyloid precursor protein modulates Nav1.6 sodium channel currents through a Go-coupled JNK pathway
Source: Sci Rep. 2016 Dec 23;6:39320. doi: 10.1038/srep39320 (PMC5180232; doi:10.1038/srep39320)
Supplement: Supplementary Information [file srep39320-s1.pdf]

Amyloid precursor protein modulates Nav1.6 sodium channel currents through a Go-coupled JNK pathway

Shao Li[1,2,3], *Xi Wang*[1], *Quan-Hong Ma*[4*], *Wu-lin Yang*[3], *Xiao-Gang Zhang*[1], *Gavin S. Dawe*[2,5,6*] *and Zhi-Cheng Xiao*[3,7*]

Supplementary Figures and Legends

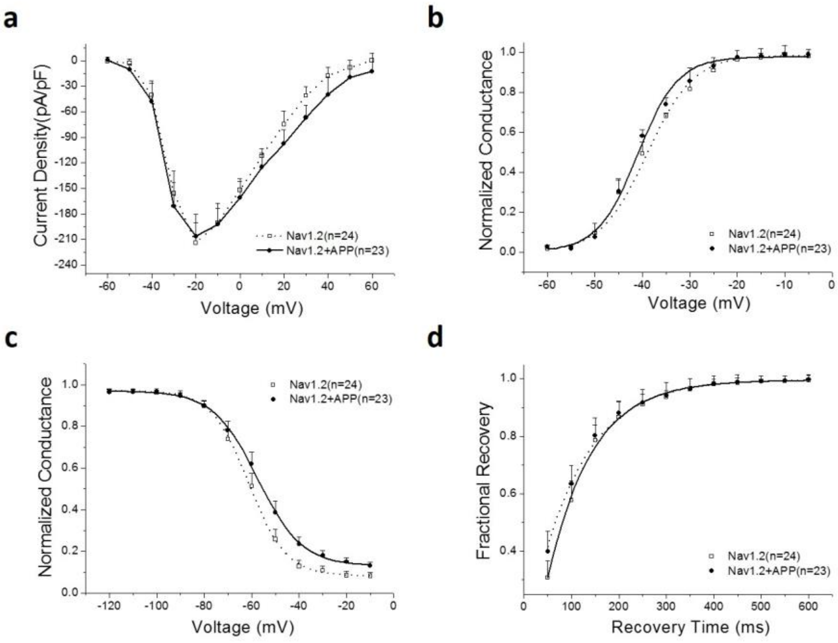

S1. Overexpression of APP did not changed sodium currents in HEK293-Nav1.2 cells.

a, Current-voltage relationships of sodium currents density in vector-(open squares) and APP-transfected HEK293-Nav1.6 cells (closed circles). pF, picofarads.

b, Activation of sodium currents in HEK293-Nav1.2 cells in vector-(open squares) and APP-transfected HEK293-Nav1.6 cells (closed circles).

c, Normalized inactivation of sodium currents in HEK293-Nav1.2 cells in vector-(open squares) and APP-transfected HEK293-Nav1.6 cells (closed circles).

d, Recovery from inactivation was measured using a two-pulse protocol with a variable interval in in HEK293-Nav1.2 cells in vector-(open squares) and APP-transfected HEK293-Nav1.6 cells (closed circles).

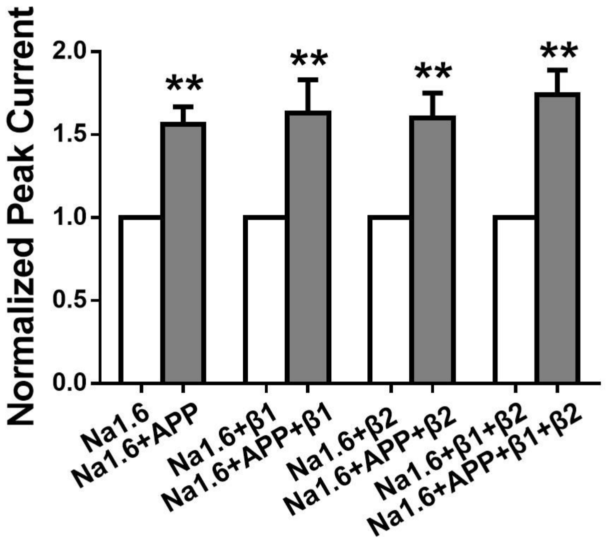

S2. Sodium peak currents in Xenopus oocytes which were injected cRNA for Nav1.6 α subunits and together with APP and β1 or β2 subunits.

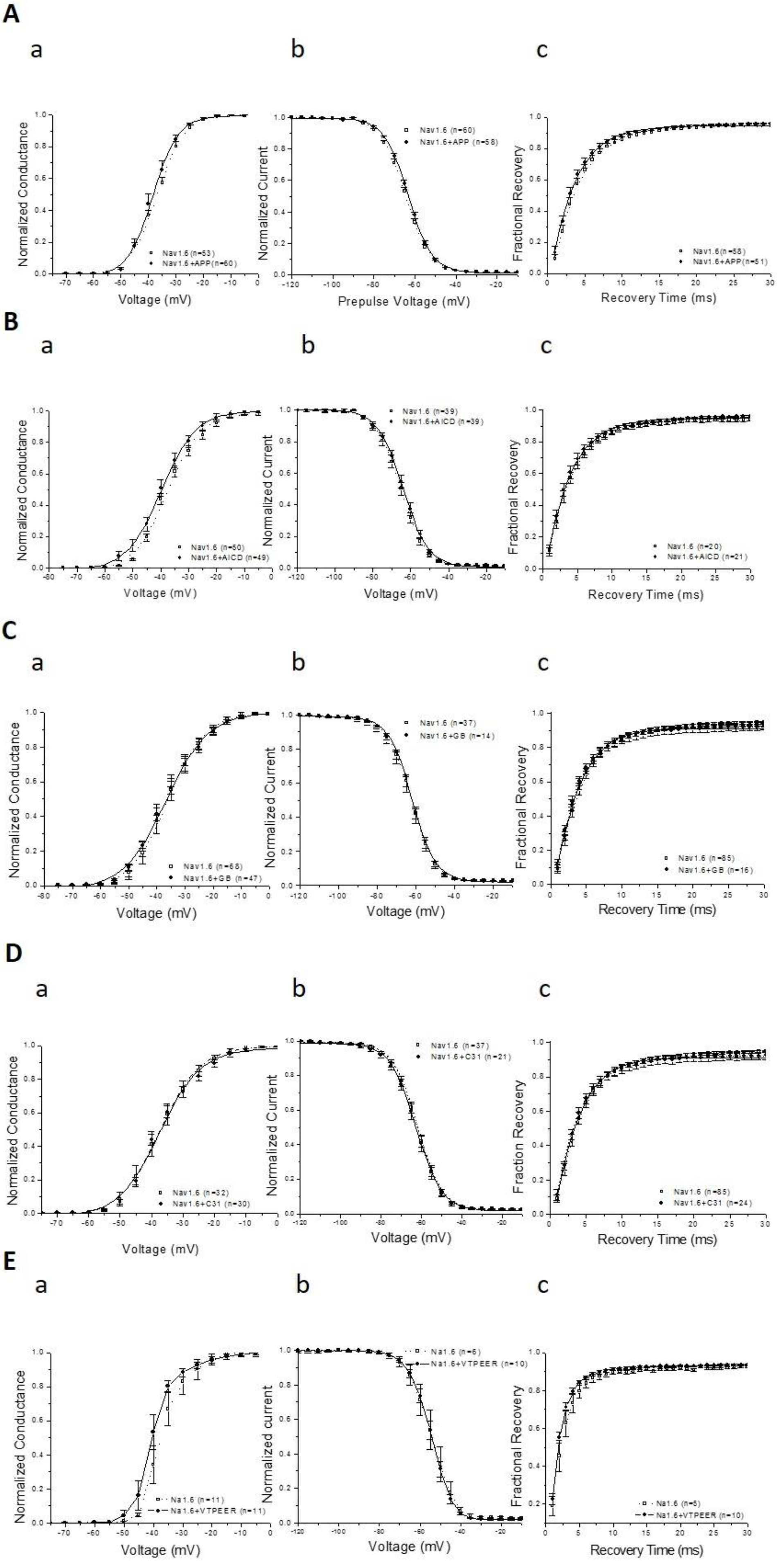

A
a
b
c
Normalized Conductance
Voltage (mV)
Nav1.6 (n=53)
Nav1.6+APP(n=60)
Normalized Current
Prepulse Voltage (mV)
Nav1.6 (n=60)
Nav1.6+APP (n=58)
Fractional Recovery
Recovery Time (ms)
Nav1.6(n=58)
Nav1.6+APP(n=51)
B
a
b
c
Normalized Conductance
Voltage (mV)
Nav1.6 (n=50)
Nav1.6+AICD (n=49)
Normalized Current
Voltage (mV)
Nav1.6 (n=39)
Nav1.6+AICD (n=39)
Fractional Recovery
Recovery Time (ms)
Nav1.6 (n=20)
Nav1.6+AICD (n=21)
C
a
b
c
Normalized Conductance
Voltage (mV)
Nav1.6 (n=68)
Nav1.6+GB (n=47)
Normalized Current
Voltage (mV)
Nav1.6 (n=37)
Nav1.6+GB (n=14)
Fractional Recovery
Recovery Time (ms)
Nav1.6 (n=85)
Nav1.6+GB (n=16)
D
a
b
c
Normalized Conductance
Voltage (mV)
Nav1.6 (n=32)
Nav1.6+C31 (n=30)
Normalized Current
Voltage (mV)
Nav1.6 (n=37)
Nav1.6+C31 (n=21)
Fraction Recovery
Recovery Time (ms)
Nav1.6 (n=85)
Nav1.6+C31 (n=24)
E
a
b
c
Normalized Conductance
Voltage (mV)
Na1.6 (n=11)
Na1.6+VTPEER (n=11)
Normalized current
Voltage (mV)
Na1.6 (n=6)
Na1.6+VTPEER (n=10)
Fractional Recovery
Recovery Time (ms)
Na1.6 (n=5)
Na1.6+VTPEER (n=10)

S3. The effects of APP, AICD, GB, C31 and VTPEER on sodium channel kinetics.

a, activation curve; **b,** inactivation curve; **c,** recovery curve.

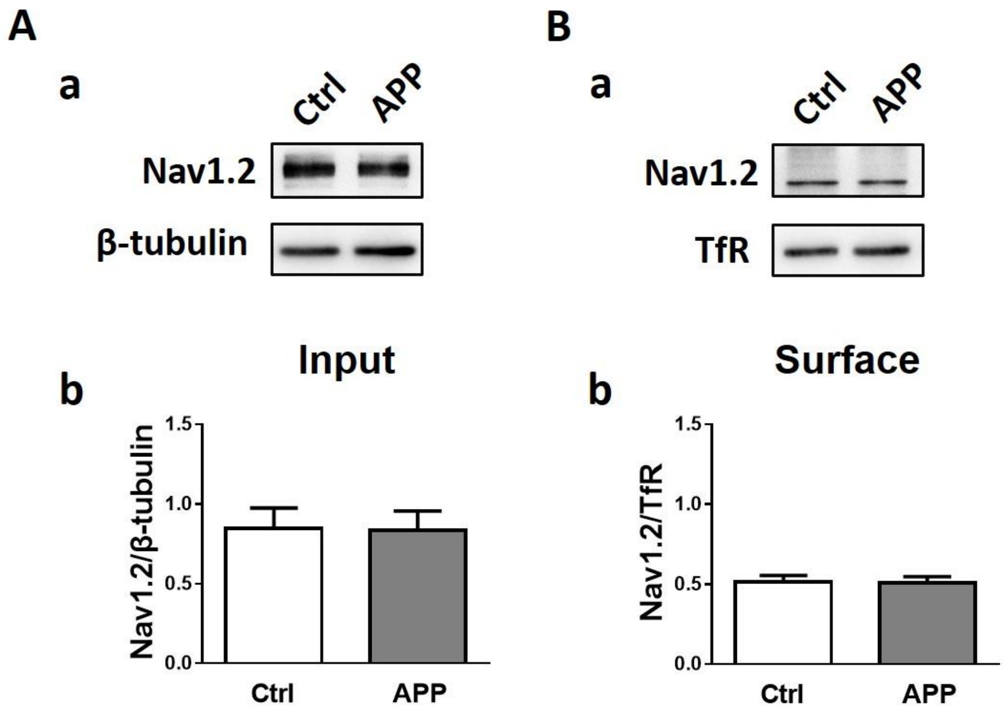

S4. The cell surface expression of Nav1.2 sodium channels in HEK293-Nav1.2 cells in vector- and APP-transfected HEK293-Nav1.2 cells.
